# Bone from Healthy Individuals and Patients with CKD Expresses the Sodium-Glucose Co-transporter-2 (SGLT2)

**DOI:** 10.1007/s00223-026-01498-7

**Published:** 2026-03-13

**Authors:** Lauter Eston Pelepenko, Luciene Machado dos Reis, Luzia Naoko Shinohara Furukawa, Rodrigo Bueno de Oliveira

**Affiliations:** 1 Faculdade de Ciências Médicas, Laboratório para o Estudo Mineral e Ósseo em Nefrologia (LEMON), Universidade Estadual de Campinas (UNICAMP), Rua Cinco de Junho, 350, Cidade Universitária Zeferino Vaz CEP, Campinas, SP 13083-877 Brazil; 2 Laboratório de Fisiopatologia Renal, Faculdade de Medicina da USP, Universidade de São Paulo, LIM 16, São Paulo, Brazil; 3 Faculdade de Ciências Médicas, Departamento de Clínica Médica (Nefrologia), Universidade Estadual de Campinas (UNICAMP), Campinas, SP Brazil

**Keywords:** Bone, Chronic kidney disease, Gliflozin, MG-63, SLC5A2, SGLT2

## Abstract

Sodium-glucose co-transporter 2 (SGLT2) inhibitors are used in type 2 diabetes mellitus management, reducing the risk of cardiovascular and renal complications. It has been stated that bone cells do not express the SGLT2 co-transporter; however, the establishment of a direct SGLT2 expression in bone and osteoblast-like cells would provide a significant advance in our understanding of direct SGLT2i effects on bone tissue from patients with metabolic bone diseases, such as chronic kidney disease or osteoporosis. SLC5A2 gene expression was investigated in osteoblast-like and renal HK-2 cells, and in human iliac crest bone samples from healthy individuals and patients with diverse stages of CKD from total RNA by real-time PCR. Additionally, SGLT2 protein qualitative expression was evaluated by Western blot in osteoblast-like cell lysates and by immunohistochemistry in bone samples from apparently healthy individuals and patients with CKD. Statistical significance was set at *p* < 0.05. SLC5A2 gene expression in osteoblast-like cells is comparable to HK-2 cells. Notably, in human bone samples, SLC5A2 was detected above threshold in both healthy individuals and patients with CKD; thus, absolute quantification of its transcript number of copies was feasible in bone samples for these conditions. SGLT2 protein was detected in osteoblast-like cells. Additionally, this protein was immunodetected in osteocytes embedded in mineralized trabecular and cortical bone samples from healthy subjects and patients with CKD. SLC5A2 gene expression and SGLT2 protein were detected in human osteoblast-like cells and bone from both healthy individuals and patients with CKD. These findings constitute an essential step toward advancing the understanding of effects, if any, of SGLT2 inhibitors on bone tissue from patients with CKD.

**Supplementary Information:**

The online version contains supplementary material available at 10.1007/s00223-026-01498-7.

## Introduction

Sodium-glucose co-transporter 2 inhibitors (SGLT2i) have gained prominence in the management of type 2 diabetes mellitus due to their ability to reduce the risk of cardiovascular and renal complications, resulting in lower mortality [1–6]. Chronic kidney disease–mineral and bone disorder (CKD-MBD) impacts the skeletal and cardiovascular systems throughout all stages of kidney disease. This systemic disorder is linked with individual and combined risk factors, including uremic toxins, mineral metabolism disturbances, and alterations in immune, endocrine, and gut functions. Consequently, these combined mechanisms increase the risk of significant bone and heart complications in these patients [7].

In 2023, approximately 788 million people aged 20 years and older were estimated to have CKD [8]. A significant proportion of these patients have CKD-MBD and develop serious skeletal negative outcomes. Bone disease represent a relevant worldwide public health issue [9] where advances in understanding its pathophysiology are still to be fully obtained [10].

Previous studies associated SGLT2i use with adverse effects on bone and an increased risk of fractures [11–16]. In this context, the FDA issued a Drug Safety Communication on September 10, 2015, warning about the risk of bone fractures and decreased bone mineral density associated with the use of SGLT2 Inhibitors [17]. Subsequent studies have not confirmed an increased risk of bone fractures nor important changes in markers of bone metabolism [18–20], and other studies even indicated potential positive effects [21].

Since then, SGLT2i effects on bone health remain an area of ongoing investigation. The use of SGLT2i has increased in different populations at high risk for metabolic bone diseases, such as patients with chronic kidney disease (CKD) and those with osteoporosis [16, 22–26]. The reasons justifying this research interest are plausible as they involve anatomical and pathophysiological aspects of bone tissue.

Bone tissue is highly vascularized, receiving 10% to 15% of the resting cardiac output [27, 28], possessing a significant vascular endothelium net, and its remodeling process is highly dependent on energy. Besides, it is widely recognized that glucose is fundamental to bone tissue metabolism and skeletal integrity, and glucose transporters are expressed in bone cells [29]. Thus, it becomes clear that understanding the relationship between SGLT2i and its possible direct effects on the skeleton is of great interest. However, the limited available evidence prevents a complete understanding of the mechanisms behind this possible interaction [14, 15].

It deserves to be highlighted that a careful review of the literature sheds light on a crucial question involving SGLT2i and bone: do bone cells express the solute carrier family 5 member 2 (SLC5A2) gene, the sodium-glucose co-transporter 2 (SGLT2) protein encoder? The evidence surrounding this issue is subject to debate. A single study [30] focusing on the SLC gene family investigated samples obtained from commercial human total RNA and cardiac-derived samples (not bone), partially addressing this question; however, even though they included bone tissue and/or only bone marrow in the investigation, their main focus was to investigate the expression of SGLT1 in human cardiomyocytes and not in bone cells.

For these reasons, different authors stated that SLC5A2/SGLT2 is not expressed by osteoblasts, osteocytes, osteoclasts, bone marrow, nor by bone tissue [14, 31–37]; these are based on data from studies including mouse calvaria osteoblasts, C3H10T1/2 mesenchymal stem cells, and MC3T3-E1 cells at various stages of differentiation [36]. Another study using a single-cell approach detected SLC5A2 expression only in bone marrow, mainly in T-cells, but also B-cells, plasma cells, macrophages, and erythroid cells are mentioned in this compendium (https://www.proteinatlas.org/ENSG00000140675-SLC5A2/single+cell). Overall, this body of evidence points out a knowledge gap, which led to a call for investigation to understand if glucose transport in bone cells is affected by treatment with gliflozins through the SGLT2 protein [38].

Clarifying these issues is an essential step toward advancing our understanding of the direct effects, if any, of SGLT2i on bone tissue from patients with metabolic bone disease, such as those with CKD and with osteoporosis. The present study aimed to document the expression of both the SLC5A2 gene and the SGLT2 protein in osteoblast-like cells and clinical bone samples from healthy individuals and patients with advanced CKD.

## Methods

### Human Bone Samples

Bone samples obtained by biopsy from apparently healthy individuals and patients with CKD were used to proceed with RNA extraction. TRIzol™ reagent (Thermo Fisher Scientific, USA) manufacturer instructions were followed for total RNA extractions. The clinical and demographic characteristics of the healthy individuals were (n = 3): 2 males (67%), with a mean age of 50 ± 3 years (deceased by trauma), and 1 female, aged 51 years (deceased by subarachnoid hemorrhage); mean serum creatinine levels were 1 ± 0 mg/dL. Bone samples from organ donors were obtained from the skeletal and muscle tissue bank of the Instituto de Ortopedia da Faculdade de Medicina da Universidade de São Paulo (BTME IOT FMUSP).

The clinical and demographic characteristics of patients with CKD (n = 10) were: 9 (93%) male patients, with a mean age of 49 ± 14 years old. CKD patients were at stages 4 and 5 (including patients under dialysis and kidney transplant). The bone biopsy samples (from iliac crests) of CKD patients were obtained from previous studies [39, 40]. Bone biopsies were performed using a trephine needle (7 mm in diameter) adapted to an electrical drill (Dewalt and Rochester bone trephine, USA), as previously described [39].

Additionally, another set of bone samples from apparently healthy subjects and patients with CKD were used for immunohistochemistry analysis. The apparently healthy individuals were female (n = 5) with a mean age of 34 ± 7 years (deceased by trauma, cerebral edema/acute drug poisoning, or cerebral hemorrhage). The patients with CKD were male (n = 6) with a mean age of 54 ± 8 years and were on hemodialysis.

Written informed consent was obtained from all patients, and the local ethics committee approved the studies [39, 40] under protocols CAAE codes 38,108,314.6.0000.5404, 45,943,115.9.0000.5404, 45,777,015.5.0000.5404, and CAPPesq 0776/11. The study was conducted in full consistency with the Declaration of Helsinki.

### Cell Line Cultures

Human osteoblast-like (MG-63) and human kidney (HK-2) cell lines were obtained from the American Type Culture Collection (ATCC CRL-1427 and -2190, USA). HK-2 served as a well-known expressor of the target SLC5A2 gene. Cells were cultivated in Dulbecco’s Modified Eagle Medium (DMEM) supplemented with 10% fetal bovine serum (FBS, Sigma Aldrich, USA), and 100 IU/mL penicillin and 100 μg/mL streptomycin (all from Gibco) in a humidified incubator with 5% carbon dioxide atmosphere at 37 °C. The supplemented media was replenished every two days for seven days. In sequence, cells were detached using 0.25% sterile-filtered trypsin–EDTA solution (Sigma Aldrich, USA) and lysed using TRIzol™ reagent. Duplicates of two biologicals were cultivated for each cell line without any treatment. These cultures served for total RNA extraction, and MG-63 lysates also for protein analysis.

### Quantitative Polymerase Chain Reaction (qPCR) Analysis

Total RNA quantification (in ng/µL) was performed using a NanoDrop™ 2000 spectrophotometer (Thermo Fisher Scientific, Waltham, MA, USA). Transcription to cDNA was performed using GoScript™ reverse transcription system (Cat A5001, Promega, USA) according to the manufacturer’s instructions, consistently using 1 µg of the total RNA. A second quantification (ng/µL) of the resulting double strand cDNA was quantified (in ng/µL) using a Qubit 2.0 Fluorometer (Qubit dsDNA BR [Broad-Range] assay buffer Thermo Fisher Scientific, Cat. No. Q32850, USA) according to the manufacturer’s protocol. In sequence, dilutions were performed to obtain cDNA sample concentrations of 3.741 ± 0.36 ng/µL.

Primers were selected using the Primer-BLAST tool (https://www.ncbi.nlm.nih.gov/tools/primer-blast) and referencing a previous study [30]. SLC5A2 sequences were aligned with the transcript variant 1 of the gene registered at the National Library of Medicine under the accession number NM_003041.4 (https://www.ncbi.nlm.nih.gov/nuccore/NM_003041.4). Preliminary real-time (qPCR) amplifications for the primer selection were performed between MG-63 and HK-2 cells cDNA to ensure a comparable expression between the human osteoblast-like cell line and the already expected [41, 42] expression of SLC5A2 by HK-2 cells. The selected SLC5A2 and the housekeeping GAPDH primers are detailed in Table 1.

**Table 1:** Primers’ data of target and housekeeping

Gene	Accession number	Product length (bp)	Forward	Reverse
h_SLC5A2 [30]	NM_003041.4	108	TTCAGTCTCCGGCATAGCAAG	CATCTCCATGGCACTCTCTGG
h_GAPDH [45]	NM_002046.7	102	CAAGAGCACAAGAGGAAGAGAG	CTACATGGCAACTGTGAGGAG

Of note, GAPDH (glyceraldehyde-3-phosphate dehydrogenase) is a widely used, highly expressed housekeeping gene in RT-qPCR analysis for internal normalization for cells lysates [43]. While popular due to its role in glycolysis, it is not always the best choice for tissue samples as its expression can vary under different conditions, making it less suitable in certain tissue-related complex contexts [44]. Here, the same GAPDH validated primer sequence [45] was used as a housekeeping for both cells and tissue lysates to observe its stability across the samples evaluated here. Also, GAPDH was used as housekeeping in other human bone tissue-related studies [39, 46].

The qPCR reactions were performed using the 7500 Real-Time PCR System with a 2X PowerUp SYBR™ Green Master Mix (Applied Biosystems, USA). All reactions were assembled strictly according to the manufacturer’s instructions using 96-well plates (MicroAmp Optical reaction plate and MicroAmp Optical adhesive film, Thermo Fisher, USA). Reaction wells (composing 20 µL) comprised 156.25 nM of each primer (forward or reverse) resuspended in 0.25 µL each, 10 µL of 2X PowerUp SYBR™ Green Master Mix, 7.5 µL of RNA/DNAse-free water, and 2 µL of the concentration-adjusted cDNA samples. Thus, the cDNA template provided was 7.481 ± 0.72 ng, which falls within the range (1–10 ng) for optimal reaction. No template control (NTC) reactions were also performed. The qPCR thermal cycling protocol consisted of an initial denaturation step at 95 °C for 10 min, followed by 40 amplification cycles of denaturation at 95 °C for 15 s and annealing/extension at 60 °C for 1 min. A dissociation stage (melting curve analysis) was added to verify amplicon specificity.

Independent assays, one for relative (2^ − ΔΔCt) and another for absolute quantification, were performed to ensure robust and complementary gene expression quantitative analysis. The ΔΔCt method assay was used for the normalization against GAPDH expression between cell lines, and intra-condition between bone samples from healthy individuals and patients with CKD. Besides, the absolute quantification assay was used to estimate the transcript copy numbers using a standard curve derived from four serial dilutions (1/1000, 1/10000, 1/100000 and 1/1000000 [or log^10^ -3, -4, -5, and -6, respectively]) from the experimental controls’ (HK-2 cells and healthy individuals bone samples) product template concentrations after amplification for 40 cycles. Additionally, internal controls also using pools from biologicals of HK-2 cells and healthy bone were adopted for this assay to corroborate the results from unknown samples. Linear regressions were then used to determine the reaction slope/efficiency, and to estimate subsequent threshold cycles and the number of copies of unknown tested samples [47, 48]. The base pairs length molecular weight of each primer GAPDH and SLC5A2, multiplied by 660 g/mol/bp (constant for dsDNA) [49] was also equated for the calculations [50].

### Western Blot Analysis for SGLT2 Detection in Osteoblast-like MG-63 Cells

Protein lysates from MG-63 cell cultures were extracted from 6-well plates (quadruplicate of biologicals cultivated in different assay plates) at full confluence using a radioimmunoprecipitation assay (RIPA) buffer. This buffer was supplemented with a protease inhibitor cocktail (PIC, Merck Sigma REF 11873580001, Complete, EDTA free, USA), 0.1 M phenylmethylsulphonyl fluoride (PMSF, Sigma P7626, USA), and 0.1 M sodium orthovanadate (Sigma S6508, USA). The total protein concentration was determined using a detergent-compatible kit (Bio-Rad DC Protein Assay kit, USA), following the manufacturer’s instructions, in µg/µL. For the analysis, 50 µg of total protein from each sample was diluted in ultra-pure water to a final volume of 15 µL, and 5 µL of 4 × Laemmli (1,610,747, Bio-Rad, USA) sample buffer added. These samples were then denatured at 95 °C for 5 min and separated by gel electrophoresis. Following electrophoresis, transfer was performed using a wet transfer system with a buffer composed of 25 mM Tris, 192 mM glycine, and 20% ethanol, at a constant voltage of 60 V for 120 min. Membranes were blocked in a solution of 2% bovine serum albumin (BSA, Goldbio, 9048–46-8, USA) in tris-buffered saline with 0.1% Tween 20 (20,605, ICI America Inc., USA) (TBST) for 30 min. Washing steps were performed four times for 5 min each with TBST.

For protein detection, membranes were incubated overnight at 4°C with the primary antibody anti-SGLT2 Abcam ab37296 diluted 1:1000 diluted in 5% BSA in TBST. Also, anti-GAPDH (glyceraldehyde-3-phosphate dehydrogenase) Cell Signaling 5174 diluted 1:1000 in 5% BSA in TBST, was incubated for one hour at room temperature. In sequence, secondary incubation was performed with horseradish peroxidase (HRP)-conjugated anti-rabbit Cell Signaling 5174 diluted 1:10,000 in 5% BSA, for 1 h at room temperature. Protein bands were visualized using the ECL Prime Western Blotting Detection Reagent (Cytiva, Amersham, USA) using chemiluminescent signal using the ImageQuant LAS 500 (GE Healthcare, Japan) imaging system in ‘incremental’ mode. The original resulting images were exported. For subsequent probing, the membrane was stripped in a stripping buffer (glycine, SDS, pH 2.2) for 30 min at room temperature.

### Immunohistochemistry Analysis for SGLT2 Colocalization in Human Bone

For the immunohistochemical qualitative analysis, the calcified bone slides (5 µm thickness) were obtained based on a previous study method [51] and processed to remove methyl methacrylate by immersion in a 1:1 xylene/chloroform solution for 30 min under slow agitation. Bone tissue slides were subsequently rehydrated through a series of graded ethanol baths (100%, 96%, 70%, and 50%), followed by a 5-min wash using 1X PBS. A semi-decalcification step was performed in 1% acetic acid for 15 min. To block endogenous peroxidase activity, slides were incubated in 30% hydrogen peroxide with methanol for 30 min in the dark, followed by washes in distilled water and 1X PBS buffer. Tissue permeabilization was achieved with 0.1% Tween 20 in PBS for 30 min. Non-specific binding was blocked using an Avidin/Biotin Blocking Kit (1:100, Vector, USA) and a subsequent 30-min incubation.

Sections were incubated with the primary antibody (anti-SGLT2 Abcam ab37296 diluted 1:20) overnight at 4°C. Then, after washing, slides were incubated with the biotinylated secondary antibody (horse anti-rabbit, Vector BA1000, 1:1000 in BSA 0,5%) for 45 min at room temperature.

The signal was evaluated after incubation for 20 min with a chromogenic substrate (0.015% hydrogen peroxide and 0.02% 3-amino-9-ethylcarbazole dye in 50 mM acetate buffer), and the sections were counterstained with Mayer’s hemalumber solution (Merck, Hohenbrunn, Germany) for approximately 2 min and mounted using glycerol gelatine. Images were obtained in different magnifications (10 to 40x, Nikon, eclipse 50i, Japan) along with scale bars. Image adjustments were restricted to brightness and contrast, with a maximum variation range of 20% and image temperature was adjusted between 5900 and 7200 K. No further or partial image adjustments were applied for the represented images.

## Statistics

Analyses of SLC5A2 gene expression had a statistical significance set at bicaudal *p* < 0.05 and were performed using GraphPad Prism software (version 10.1.1; GraphPad Software, San Diego, CA, USA). Data distribution was assessed using the Shapiro–Wilk test to confirm data normality. For simple comparisons, unpaired t-test or Mann–Whitney tests were used. One-way ANOVA followed by Tukey or Dunn post hoc tests was used for multiple comparisons. Linear regressions were performed using ‘xy’ plotting, using the same software. Data on SGLT2 protein detection by Western blot in osteoblast-like cells or by immunohistochemistry in bone samples are qualitatively described.

## Results

### Primer Selection

**Fig. 1:**
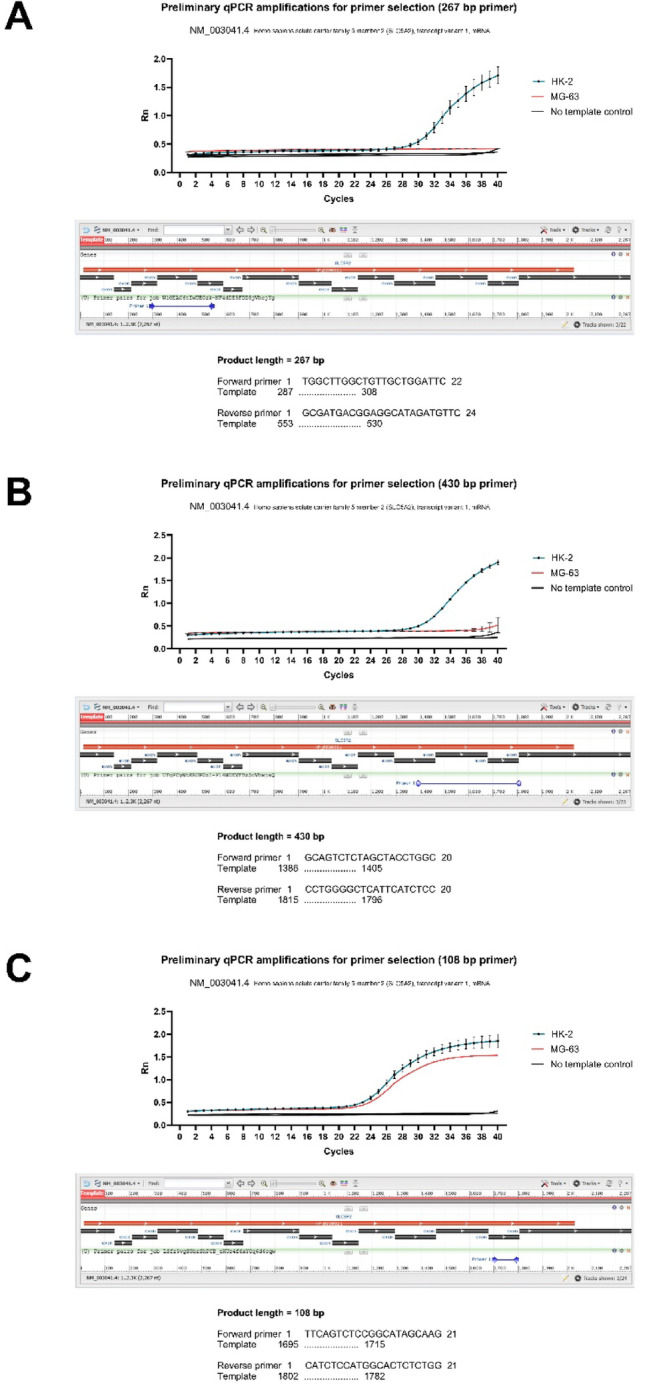
Preliminary qPCR amplification for primer selection. **A** and **B** Unsuccessful preliminary qPCR amplification using 267 and 430 bp primer pairs for SLC5A2 for MG-63 cells, respectively. The amplification plot displays the performance designed to amplify as a product of the human SLC5A2 transcript (NM_003041.4), with its specific binding sites shown below. While the primers yielded a clear amplification signal in the positive control HK-2 renal cells, no detectable expression was observed in the osteoblast-like MG-63 cell line. Due to the lack of amplification in MG-63 cells, these primer sets were deemed unsuitable for the study. (**C**) Successful validation of the selected 108 bp primer for SLC5A2. This primer pair shows robust and comparable amplification in both MG-63 and HK-2 cell lines between 22 and 24 cycles, confirming its suitability for subsequent comparative gene expression analysis by qPCR

Preliminary qPCR amplification reactions for primers selected within the SLC5A2 sequence (NM_003041.4) that culminated in the selection of a pair that expressed in both cell lines are exhibited in Fig. 1. Two primer pairs failed to yield satisfactory amplification: the first, targeting a 267 bp product (sequence positions 287–553), and the second, amplifying a 430 bp fragment (sequence positions 1386–1815), showed poor or no expression in the osteoblast-like cell line. In contrast, the third analyzed primer pair, designed to amplify a 108 bp product (sequence positions 1695–1802), consistently produced a clear and specific product in both osteoblast-like and HK-2 cell lines between 22 and 24 cycles, and was thus selected for further expression analysis also in human bone samples. It is noteworthy that all three SLC5A2 primer pairs successfully amplified their respective targets in HK-2 cells samples, as expected. Of note, the 267 bp and 430 bp primers produced clear amplification in HK-2 cells, but not in osteoblast-like cells, confirming their target specificity for this kidney cell line.

### Quantitative Polymerase Chain Reaction (qPCR) Analysis

**Fig. 2:**
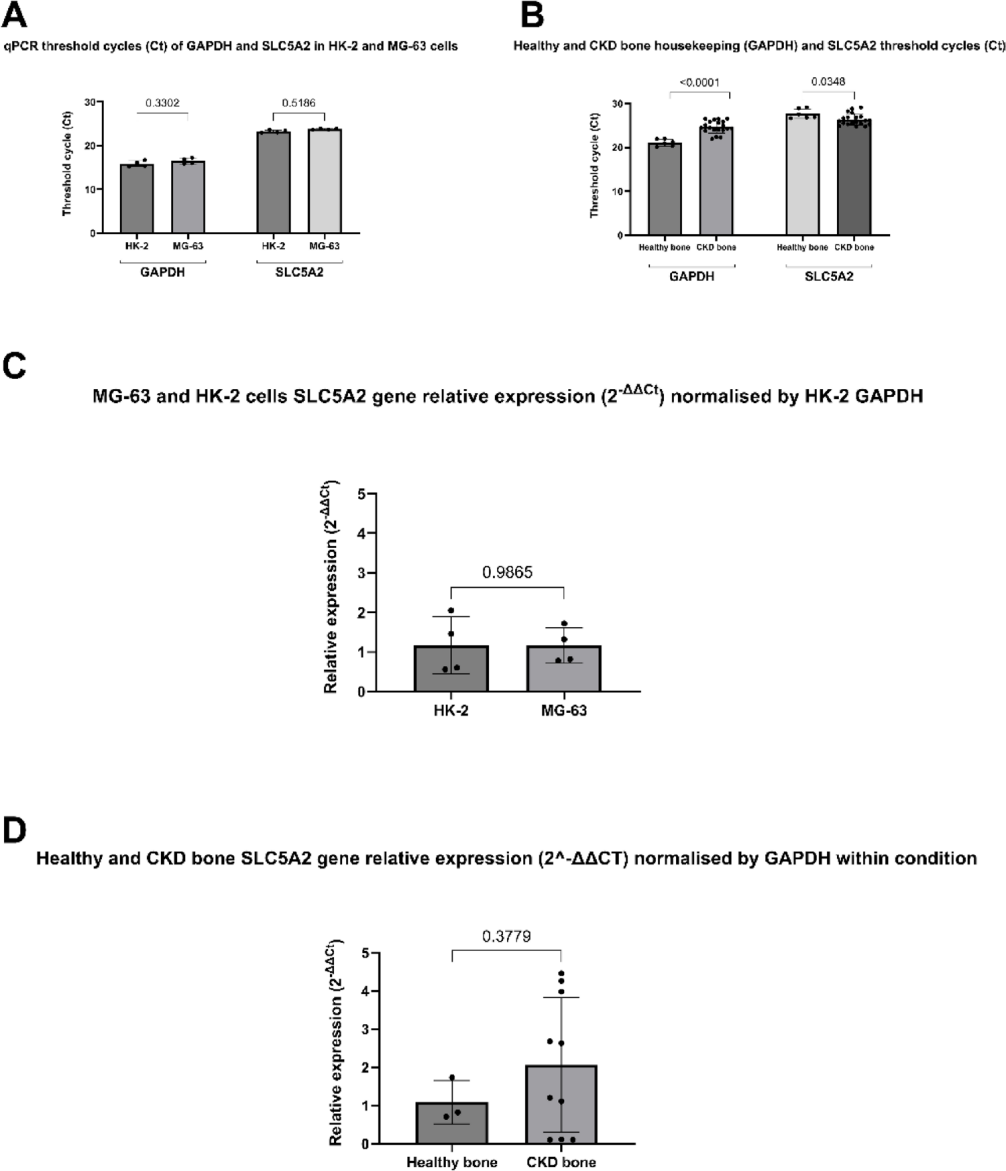
Relative quantification of SLC5A2 gene expression. **A** Raw Ct values of two biologicals (in duplicates) showing comparable SLC5A2 expression in MG-63 and HK-2 cells; for the first time, the SLC5A2 expression in osteoblast-like MG-63 cells is confirmed; **B** Raw Ct values of three healthy individuals bone samples and ten bone samples from patients with CKD (in duplicates) indicating that the SLC5A2 expression is lower in bone samples from patients with CKD. After ΔΔCt normalization by GAPDH, no significant difference is seen between **C** cell lines (two biologicals in duplicates), or between **D** bone samples (mean of duplicates) from healthy subjects and patients with CKD. GAPDH variation in expression (above 3.5 Cts) prevented a suitable normalization of SLC5A2 expression in healthy subjects versus patients with CKD; thus, each condition was normalized to its own GAPDH expression for representation purposes

The ΔΔCt method analysis is illustrated in Fig. 2 (A and B), presenting qPCR threshold cycle (Ct) data that compare the expression of GAPDH and SLC5A2 under two different biological contexts. For the cell comparisons, Ct values of GAPDH and SLC5A2 in HK-2 and osteoblast-like cell lines are demonstrated. As expected, GAPDH expression was comparable between the two cell lines (*p* = 0.3302), confirming its suitability as a housekeeping control for these cell lines. SLC5A2 expression was also detected in both cell types, with no significant difference in Ct values (*p* = 0.5186), suggesting comparable transcriptional levels. Figure 2C shows the SLC5A2 expression in the HK-2 and osteoblast-like cell lines, normalized to GAPDH in HK-2 cells. The relative expression levels were also comparable between the two cell types (*p* = 0.9865). This suggests that, despite MG-63 being an osteoblast-like cell line and HK-2 being kidney-derived (where SLC5A2 is typically enriched), the gene is expressed at similar levels under in vitro culture conditions.

Regarding bone samples analysis, SLC5A2 amplification profile occurred at lower Ct values in bone from patients with CKD (*p* = 0.0348), indicating increased expression in comparison with bone samples from healthy individuals. However, the GAPDH expression was significantly lower in CKD bone, as indicated by elevated Ct values (*p* < 0.0001), suggesting lower expression and potential variability of GAPDH as a housekeeping (reference) gene in CKD-altered bone. Considering the ΔΔCt analysis of bone samples from patients with CKD (Fig. 2D), remarkable differences (above 3.5 Cts) between conditions’ GAPDH expression prevented a suitable normalization of SLC5A2 expression in healthy individuals versus patients with CKD; thus, each condition was normalized to its own GAPDH expression.

**Fig. 3:**
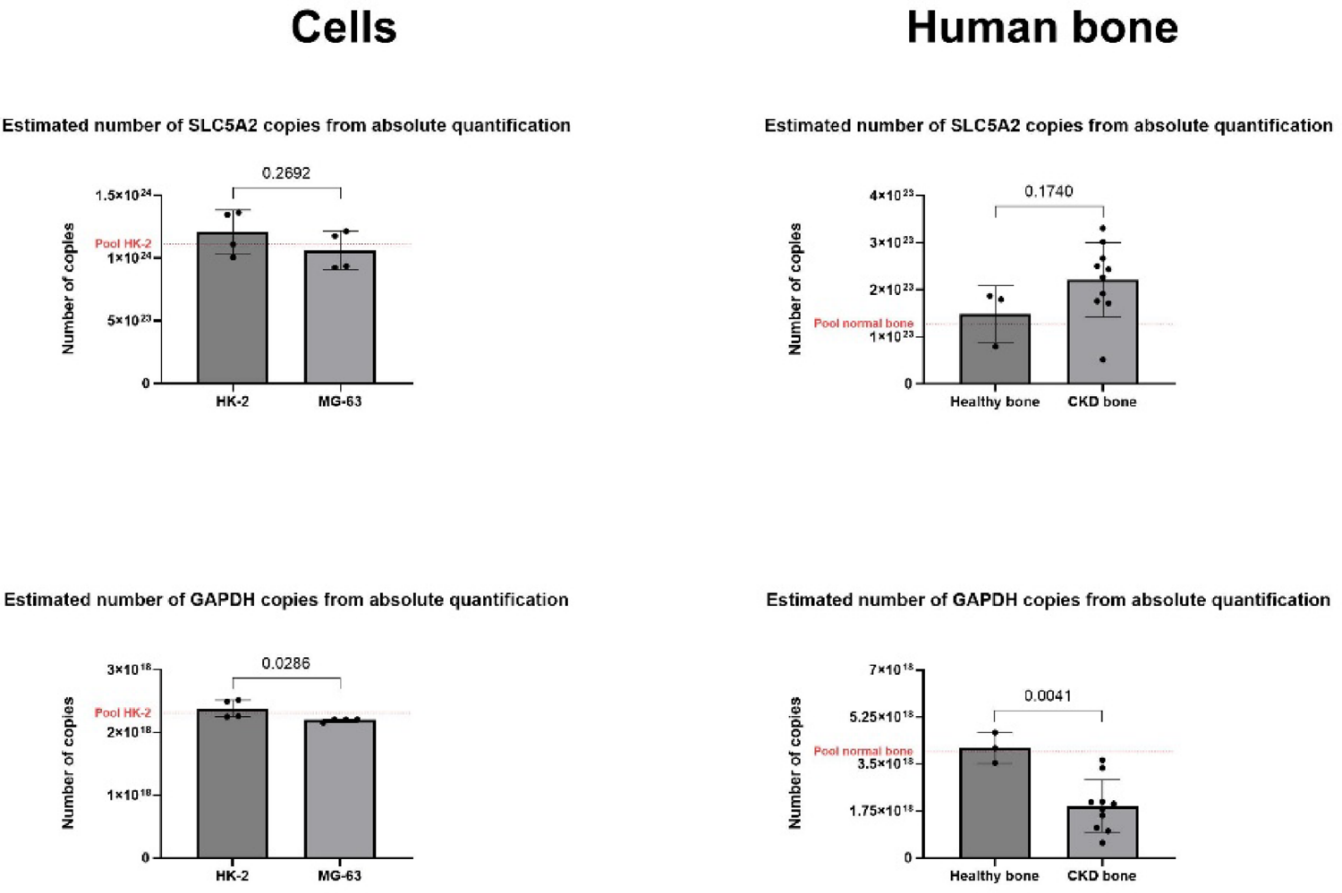
Absolute quantification of SLC5A2 and GAPDH transcript copy numbers in cells (two biologicals in duplicates) and human bone (mean of duplicates from three healthy individuals’ bone and ten samples from patients with CKD) after 40 cycles. The use of standard curves and linear regressions using dilutions in log^10^ -3, -4, -5, and -6 from experimental controls (HK-2 cells and apparently healthy individuals´ bone samples) templates allowed the estimation of an unknown number of copies for each primer individually. This analysis confirms comparable absolute SLC5A2 transcript levels between HK-2 and osteoblast-like MG-63 cells, while GAPDH levels were modestly lower in MG-63 cells. In human bone tissue, a non-significant trend towards higher SLC5A2 copies was observed in samples from patients with chronic kidney disease (CKD) compared to samples from healthy subjects. Critically, GAPDH copy numbers were significantly reduced in CKD bone, demonstrating its instability as a reference gene in this pathology and reinforcing the value of absolute quantification for accurate expression analysis while analyzing bone samples. Additionally, internal controls also using pools from biologicals of HK-2 cells and healthy bone (red dashed line indicated within each ‘y’ axis) were adopted as an extra sample (in duplicates) to corroborate the unknown individual sample results; this reference obtained its number of copies within the range of their tested biologicals

The absolute quantification assay is represented in Fig. 3 for SLC5A2 and GAPDH transcript copy numbers. Each graph also features a red dashed line labeled “pool,” representing the pooled control samples (HK-2 cells or bone from healthy subjects), which served as an internal control for amplifications. The absolute quantification revealed a comparable number of SLC5A2 transcript copies in both HK-2 and osteoblast-like cells (*p* = 0.2692). Both cell types expressed SLC5A2, indicating comparable gene activation under baseline culture conditions. Absolute quantification of SLC5A2 in human bone revealed transcript levels in bone samples from patients with CKD, with a higher mean number of SLC5A2 copies than in bone from apparently healthy subjects, although without statistical significance (*p* = 0.1740). Notably, most bone samples from patients with CKD clustered near 1.5-fold above the red dashed line (bone pool from the apparently healthy subjects [1.278 × 10^23^]). GAPDH copy numbers were significantly lower in bone samples from patients with CKD compared to bone from healthy subjects (*p* = 0.0041), confirming impaired or variable expression of this commonly used reference gene. Bone samples from healthy subjects exhibited a consistent range of GAPDH transcripts, clustering around the red dashed pool line. Bone samples from patients with CKD fell markedly below this reference, with most values between 1.75 × 10^18^ and 3 × 10^18^ copies.

### Western Blot Analysis for SGLT2 Detection in Osteoblast-like MG-63 Cells

**Fig. 4:**
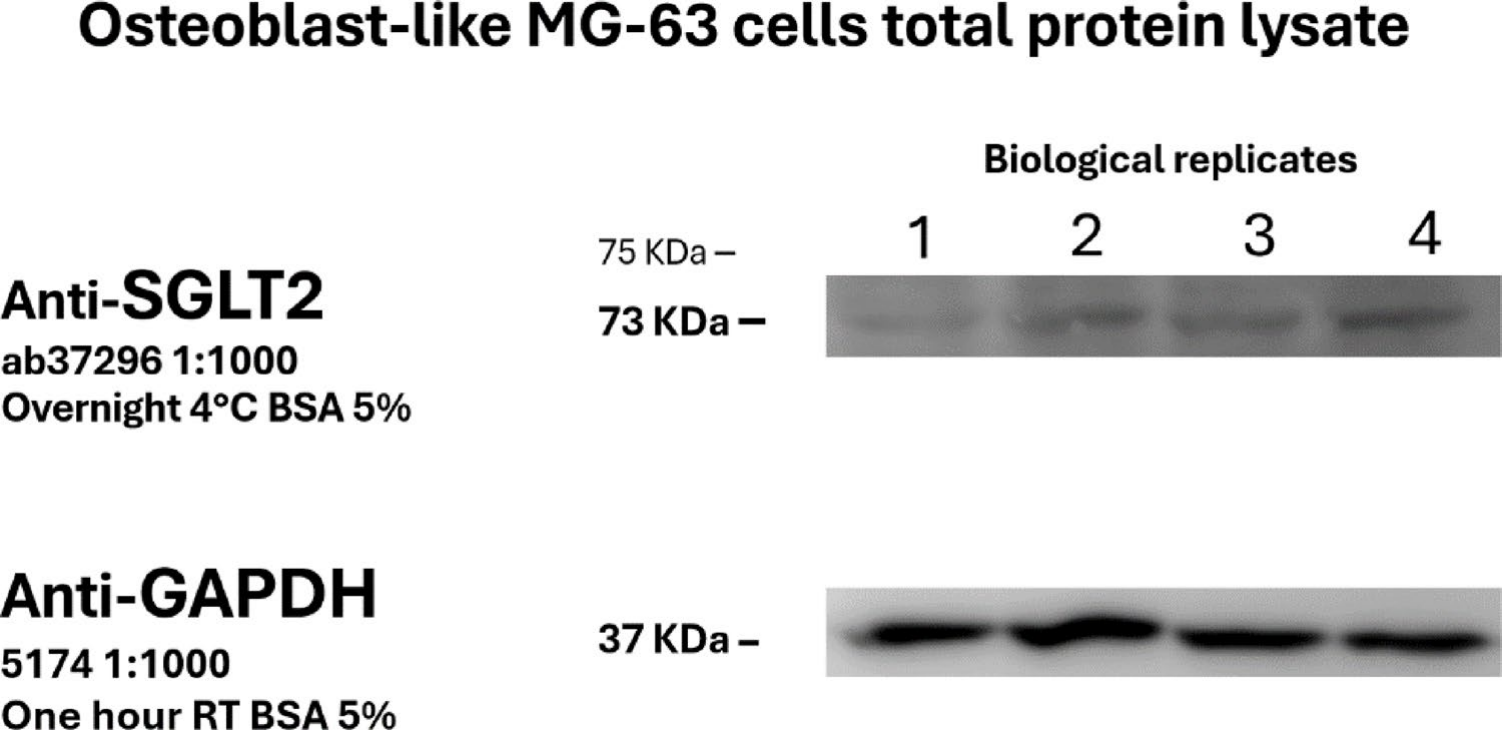
Western blot analysis of the expression of SGLT2 in four distinct biological replicates of MG-63 osteoblast-like cells. A specific band corresponding to SGLT2 was successfully detected at its predicted molecular weight of approximately 73 kDa in all four samples. Housekeeping protein GAPDH bands are uniformly observed at the expected size of 37 kDa

Western blot analysis of osteoblast-like cells lysates performed to confirm the expression of SGLT2 in four distinct biological replicates is shown in Fig. 4. A specific band corresponding to SGLT2 was successfully detected at its predicted molecular weight of approximately 73 kDa in all four biologicals. Subsequent probing for housekeeping protein GAPDH showed uniform bands at the expected size of ~ 37 kDa. The consistent intensity of the GAPDH bands confirms an equal amount of loaded protein, validating the detection of SGLT2. This result confirms the presence of the SGLT2 protein in the analyzed osteoblast-like MG-63 samples.

### Immunohistochemistry Analysis for SGLT2 Colocalization in Human Bone

**Fig. 5:**
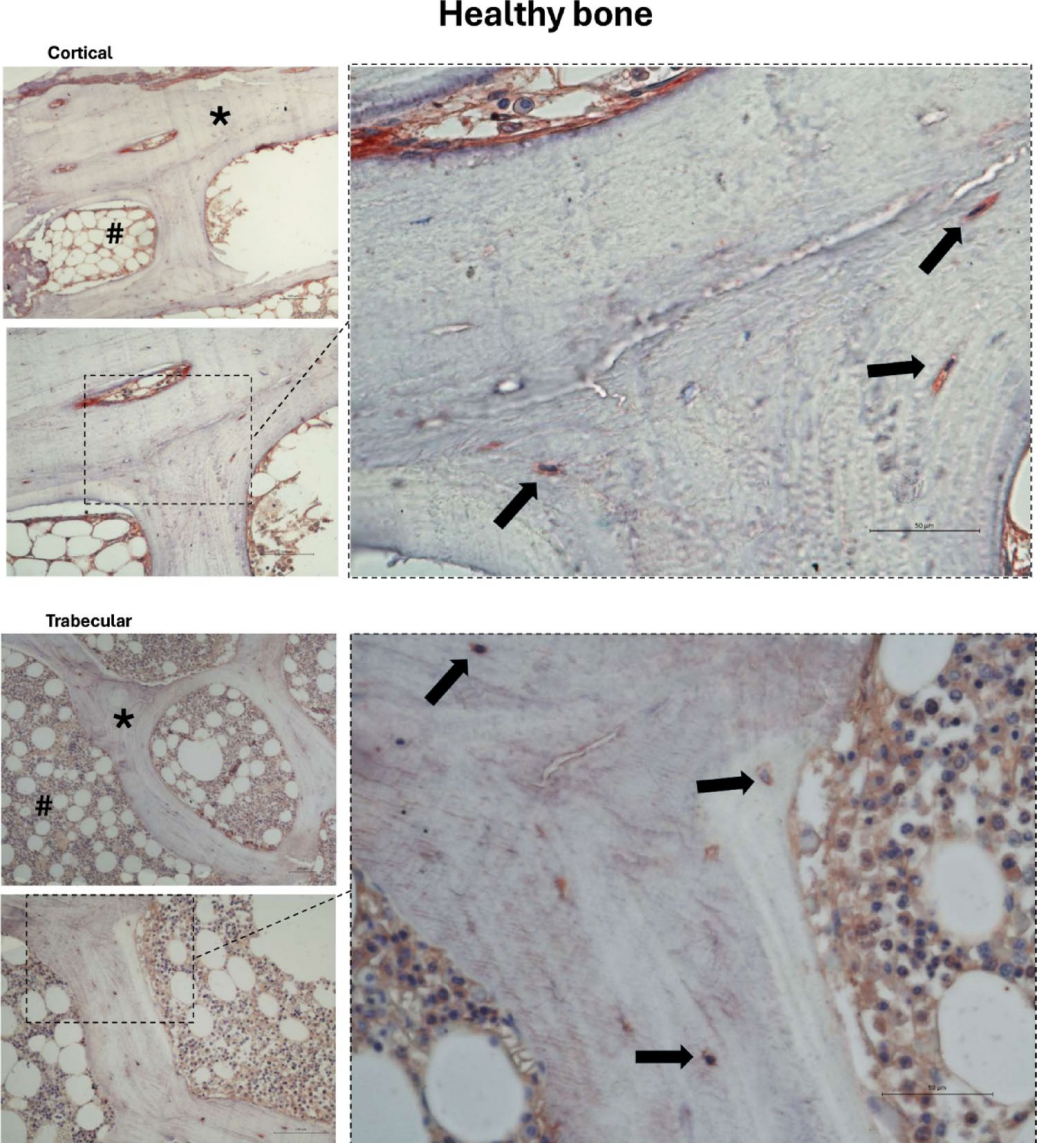
Representative images indicating the expression of SGLT2 in mineralized bone tissue from healthy subjects in cortical and trabecular bone. Immunodetection of SGLT2 co-transporter is visible in osteocytes (arrows) of trabecular and cortical bone. Healthy individuals are represented with a mean age of 34 ± 7 years. Symbols: * cortical (up) and trabecular (down) bone images; **#** bone marrow

**Fig. 6:**
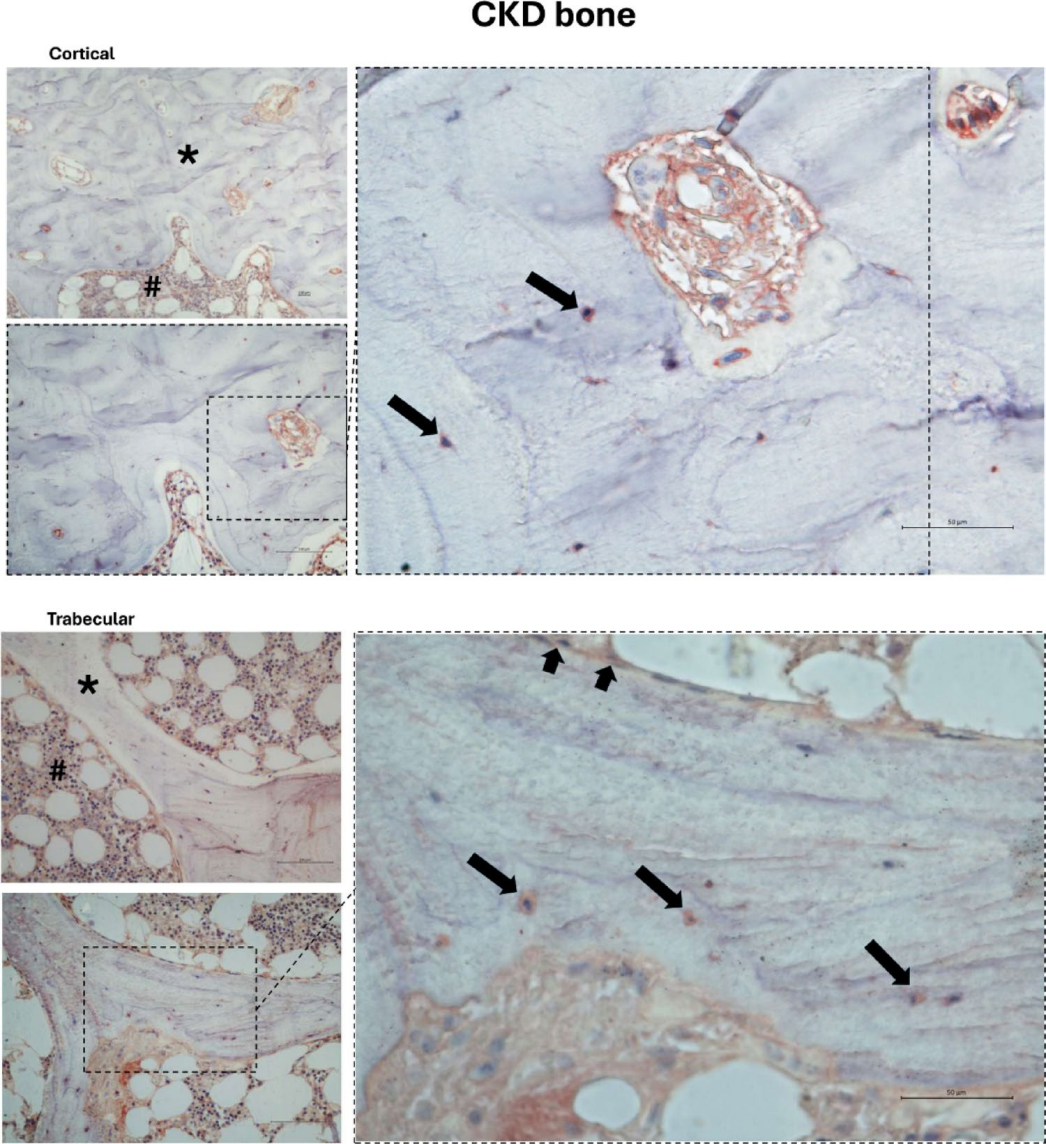
Representative images indicating the expression of SGLT2 in clinical bone biopsies from patients with CKD in cortical and trabecular bone. Immunodetection of SGLT2 co-transporter is visible in osteocytes (dark-filled arrows) and lining cells (short dark-filled arrows) of trabecular and osteocytes of cortical bone. CKD represents patients in hemodialysis. Symbols: * cortical (up) and trabecular (down) bone images; **#** bone marrow

**Fig. 7:**
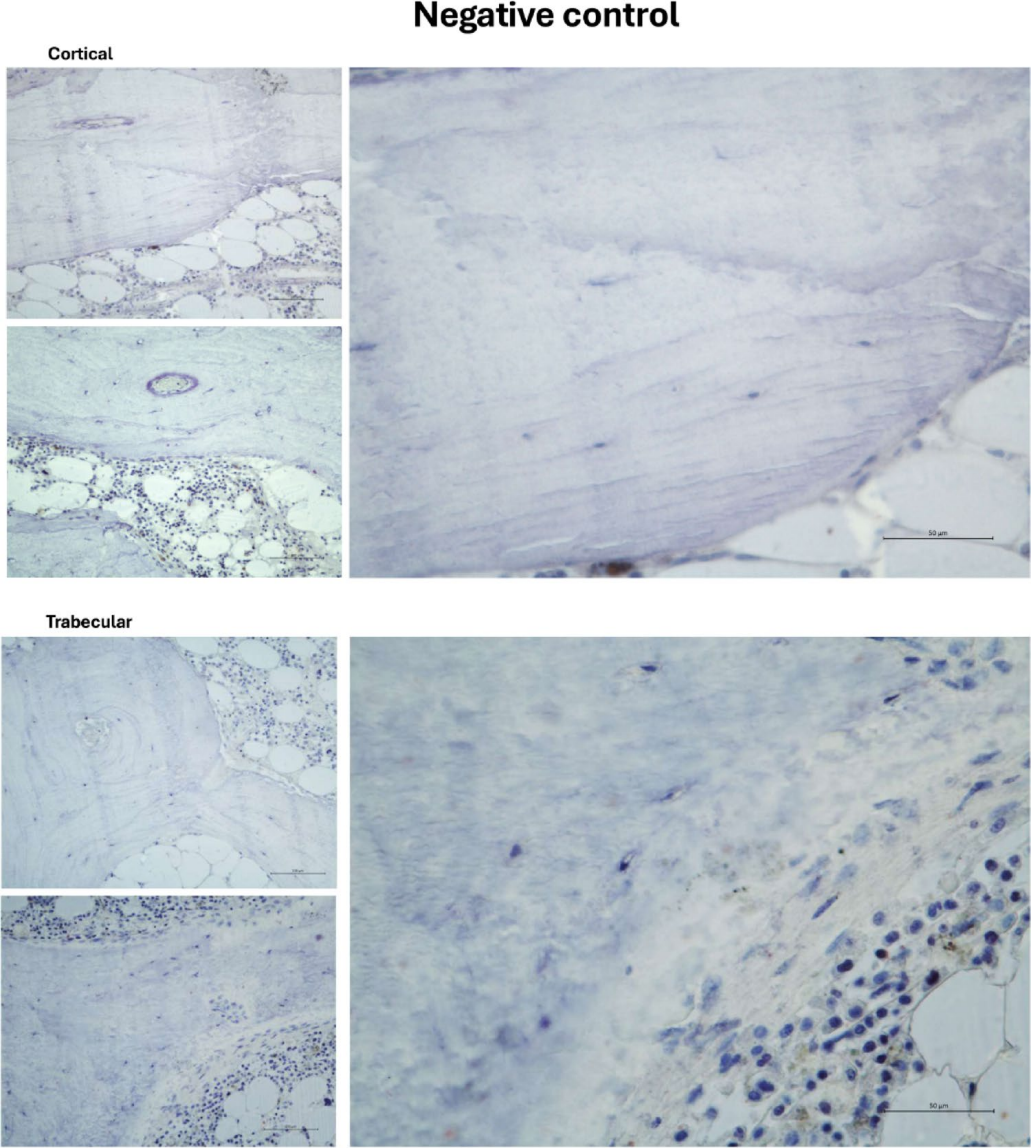
Representative negative control without SGLT2 antibody incubation, but counterstained. Similar backgrounds are visible in the images provided in main file; here, without immunodetection of SGLT2

SGLT2 consistently stained in human bone by immunohistochemistry on clinical bone biopsies, as represented in Figs. 5 and 6 for healthy subjects and patients with CKD, respectively. The positive markings provide the first direct evidence of SGLT2 expression within mineralized bone tissue, in trabecular as well as in cortical bone. In healthy individuals, high-magnification images revealed specific SGLT2 staining in osteocytes along mineralized trabecular surfaces, as well as within osteocytes embedded in the cortical bone. In bone samples from patients with CKD, SGLT2 immunostainings were similarly identified in osteocytes and lining cells of the trabecular bone, and within the cortical osteocytes. A qualitative analysis suggested the SGLT2 immunostaining distribution pattern was more evident in bone biopsies from patients with CKD. These findings collectively demonstrate that the SGLT2 co-transporter is fundamentally expressed in key bone cell populations across different bone compartments. Negative control images of slides without antibody during incubation are represented in Fig. 7.

## Discussion

The findings from this study demonstrate the expression of the SLC5A2 gene and SGLT2 protein in osteoblast-like cells and bone from healthy individuals, and from patients with advanced CKD. This new data contrasts with previous assumptions [14, 31–34, 36, 38], claiming that these are not expressed in osteoblasts or osteocytes. The present study showed amplification of SLC5A2 (NM_003041.4) and localized SGLT2 immunohistochemical staining in mineralized bone tissue.

Primer validation using the 108 bp primer pair confirmed successful amplification in both cell types (MG-63 and HK-2 cells), indicating that SLC5A2 transcripts are present and detectable. Likewise, SLC5A2 was detected in healthy bone samples, and those from patients with CKD, consistently exhibiting copy numbers above threshold, validating its transcriptional presence. Notably, bone samples from patients with CKD showed a trend toward increased SLC5A2 levels, although this was not statistically significant, this might suggest a potential disease-related regulatory mechanism.

To the best of our knowledge, no previous studies have compared these two cell lines regarding SLC5A2 gene expression. The primer pair used here was previously employed in a study analyzing commercial total mRNA samples [30]. This previous study focused on the SLC gene family, especially in cardiac-derived samples, to document the SGLT1 expression. It is rather unclear whether the authors investigated whole bone tissue or only bone marrow samples in their study, although their focus of investigation was the expression of SGLT1 in human cardiomyocytes, not bone cells. In our opinion, this approach generated insufficient evidence for bone cells regarding the specific SLC5A2/SGLT2 expression. Moreover, this finding has never been confirmed by subsequent studies. It is noteworthy that another study aimed to amplify SGLT2 in mouse-related samples and reported in their supplementary material amplification solely in kidney samples [36]; this study highlight the great interest on investigate bone-related expression towards this marker.

A meta-analysis of 25 studies [14] published in 2024 concluded that SGLT2i may negatively affect bone metabolism by increasing serum PTH, CTX, and decreasing serum ALP, but had no significant effect on bone mineral density in patients with type 2 diabetes compared to placebo; however, the mechanistic or direct expression in bone was never investigated. A subsequent study [31] published in 2025 stated that SGLT2 is not expressed in osteoblasts or osteocytes and investigated genetically heterogeneous UM-HET3 mice reporting bone tissue metabolome and bone marrow RNA sequencing indicative of sex-specific systemic and skeletal responses, metabolic changes in cortical bone tissue, and bone turnover in sex- and age-dependent manners; however, also without a direct genetic expression approach by bone cells. Other review studies [32–35] on the subject also underscored this knowledge gap of direct SGLT2 expression by bone cells.

Here, the investigation documented the expression of the targeted segment of the NM_003041.4 sequence, allowing comparisons. Although surprising, SLC5A2 expression was found to be similar in proximal tubular kidney cells and osteoblast-like cells for this specific primer pair. As shown in the preliminary primer selection in Fig. 1, HK-2 cells expression was yielded in multiple sites of the NM_003041.4 sequence. It is also noteworthy that the resulting amplicon size should ideally be kept between 70 and 200 bp, which is crucial for accurate quantification in qPCR [52].

The absolute quantification data provide a diverse quantitative insight into the gene expression patterns observed through Ct and relative quantification methods [53]. In the cultured cells, both SLC5A2 and GAPDH were robustly expressed. In human bone, however, expression patterns become more variable. While SLC5A2 expression tended to be higher in bone samples from patients with CKD, the difference remained statistically not significant, possibly due to patient heterogeneity or differential tissue expression. In contrast, GAPDH showed a clear and significant reduction in bone samples from patients with CKD, which raises substantial doubts on its reliability as a reference gene in diseased bone tissue. Previous studies have noted that GAPDH expression can vary across tissues, cell types, and experimental conditions, making it a rather unreliable reference gene in certain situations [44, 54, 55]. Altogether, these findings stress the importance of validating reference genes for cautious interpretation of normalization strategies in CKD-bone research.

The human osteoblast-like cell line MG-63 is a representative cell for in vitro bone research [56, 57]. To date, no previous studies have investigated this cell line with the gene SLC5A2 nor its encoded protein, SGLT2. The immortalized human kidney epithelial cell line (HK-2) knowingly expresses the SLC5A2 gene [41, 42], thus serving here as a positive comparison for this targeted gene expression. The findings of this study also provide compelling evidence for the protein expression of SGLT2 in osteoblast-like MG-63 cell cultures. A key strength of this investigation is the demonstration of this expression at both the mRNA and protein levels. While the detection of gene transcripts is established solely on a genetic basis, confirming the presence of the corresponding protein represents a critical step in asserting its potential functional relevance. No previous studies investigated the SGLT2 protein expression in this osteoblast-like cell line.

Most importantly, the immunohistochemistry analysis used the same primary antibody from the Western blot analysis, aiming for consistency when detecting this protein. In this sense, the immunohistochemistry analysis confirmed the gene translation into protein and revealed its localization in mineralized trabecular and cortical bone. SGLT2 was consistently identified in osteocytes, a key regulatory cell, across both trabecular and cortical compartments in healthy individuals and patients with CKD. No previous studies have reported the immunolocalization of SGLT2 in human bone tissue. It is crucial to highlight that a brief partial decalcification was performed for bone histology processing; thus, the findings here reported are linked to a consistent processing technique [58] that preserves this structural bone calcification and provides immunostaining of specific epitopes of interest, such as SGLT2 in bone cells. The extrapolation of this analysis to fully uncalcified bone histological techniques might not be feasible.

Limitations for this study include is the ΔΔCt analysis the wide variability of GAPDH in bone samples that influenced normalization of gene expression when using the relative method. The sample size for RNA extraction from healthy subjects’ bone is low. Moreover, protein lysates of human bone were not included at this time in the protein analysis since the focus was to highlight SGTL2 expression in a cell line representative for osteoblasts, and not a lysate from bone tissue that would inevitably include other cellular and tissue elements (i.e., adipocytes, red blood cells, leucocytes, endothelial cells, and fibroblasts). However, strong aspects of the present study provided direct confirmation of protein expression, as this immunological detection goes beyond transcriptional analysis, providing preliminary evidence that SLC5A2 mRNA is translated into SGLT2 protein within this cell line, as the Western blot results demonstrate specific bands for SGLT2. Also, the use of MG-63 cells for SLC5A2 gene and SGLT2 protein differential analysis seems to be suitable when an osteoblast-like analysis separated from that of a whole tissue sample would provide. Additionally, the present study bridges the gap between gene and protein expression of SGLT2 in human bone, also providing novel evidence of SLC5A2 in CKD-altered bone.

These crucial findings serve as a pilot study for future research on SGLT2i direct effects in bone. The methodological building up here proposed include relevant aspects that were not previously combined: 1) bone sampling in different pathological conditions and healthy controls; 2) relevant cell lines for comparative expression; 3) mRNA analysis of SLC5A2 with different targeted primers allowing the primer selection of a previously investigated sequence [30]; 4) cDNA quantification ensuring similar input of sample for qPCR analysis; 5) two methods of qPCR (relative and absolute); 6) protein analysis of translated SGLT2 in a relevant cell line; and most importantly, 7) colocalization of SGLT2 expression within mineralized bone tissue by immunohistochemistry in both bone of healthy individuals and patients with CKD.

Certainly, future studies are warranted to investigate SGLT2 protein expression in bone through immunodetection and/or proteomic approaches, including confirmatory data from bone single-cell experiments. A more precise understanding of how osteoblasts, osteoclasts, and osteocytes distinctly utilize and respond to glucose modulation by SGLT2i might be critical. This path of investigation would fully clarify a potential direct mechanistic influence of SGLT2i in bone pathophysiology.

## Conclusions

In this study, SLC5A2 gene expression was detected in osteoblast-like MG-63 cells and bone samples from both healthy individuals and patients with CKD. Besides, its encoded SGLT2 protein was detected in osteoblast-like cells and immunodetected in human bone tissues from healthy individuals and patients with CKD. These findings constitute a crucial step toward advancing into a clear understanding of the potential effects, if any, of SGLT2 inhibitors in bone from patients with CKD.

## Supplementary Information

Below is the link to the electronic supplementary material.

Supplementary file1 (PDF 181 kb)

Supplementary file2 (TIF 3042 kb)

Supplementary file3 (DOCX 34 kb)

Supplementary file4 (DOCX 14 kb)

Supplementary file5 (JPG 49 kb)

Supplementary file6 (TIF 3042 kb)

Supplementary file7 (JPG 49 kb)

## Data Availability

No specific code was used in the analysis. Any additional information may be available from the lead contact upon request.
