# Supplementary material for: Bone from Healthy Individuals and Patients with CKD Expresses the Sodium-Glucose Co-transporter-2 (SGLT2)
Source: Calcif Tissue Int. 2026 Mar 13;117(1):39. doi: 10.1007/s00223-026-01498-7 (PMC12982296; doi:10.1007/s00223-026-01498-7)
Supplement: Supplementary file 1 — Supplementary file1 (PDF 181 kb) [file 223_2026_1498_MOESM1_ESM.pdf]

Supplementary information (Si)

Mycoplasma tests were assessed by RT-PCR using primers targeting the 16S rRNA gene following Young et al. (2010). To ensure that no contamination occurred during cell culturing, supernatant was collected from the cell cultures prior lysing for RNA extractions. Reactions were carried out using agarose gels following Young et al. (2010). Routine testing is performed within the laboratory cell culture facility.

Si 1 – Representative mycoplasma test for HK-2 cell culture showing negative amplification. Lane 1, 1kb ladder; lane 2, no template control; lane 3, positive control; lane 4, positive inhibition control; and, lane 5, HK-2 cell culture showing negative amplification. No cropping nor brightness/contrast adjustments were used in the original image provided below.

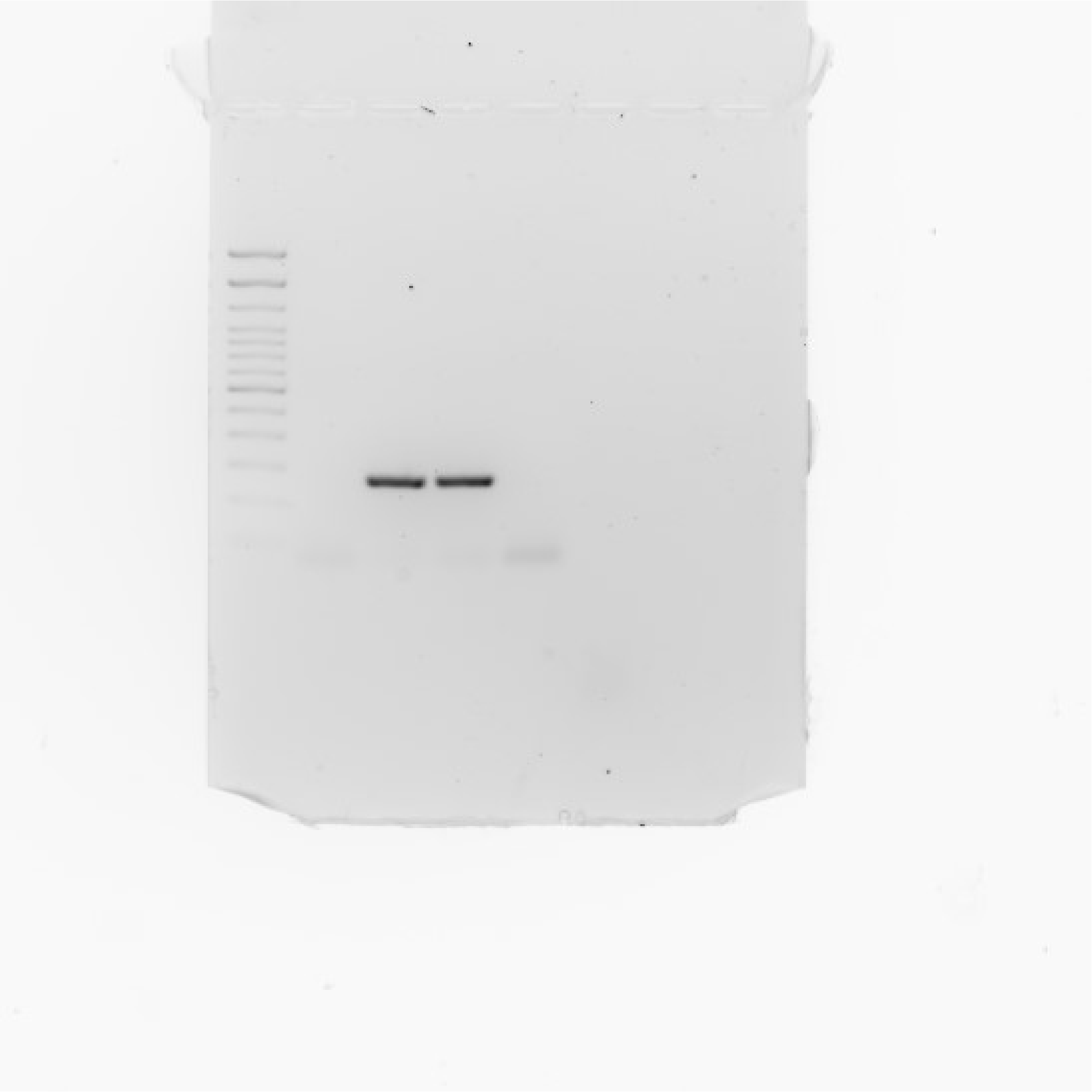

Si 2 - Representative mycoplasma test for MG-63 cell culture showing negative amplification. Lane 1, 1kb ladder; lane 2, no template control; lane 3, positive control; lane 4, positive inhibition control; and, lane 5, 6 and 7, MG-63 cell culture showing negative amplification. No cropping nor brightness/contrast adjustments were used in the original image provided below.

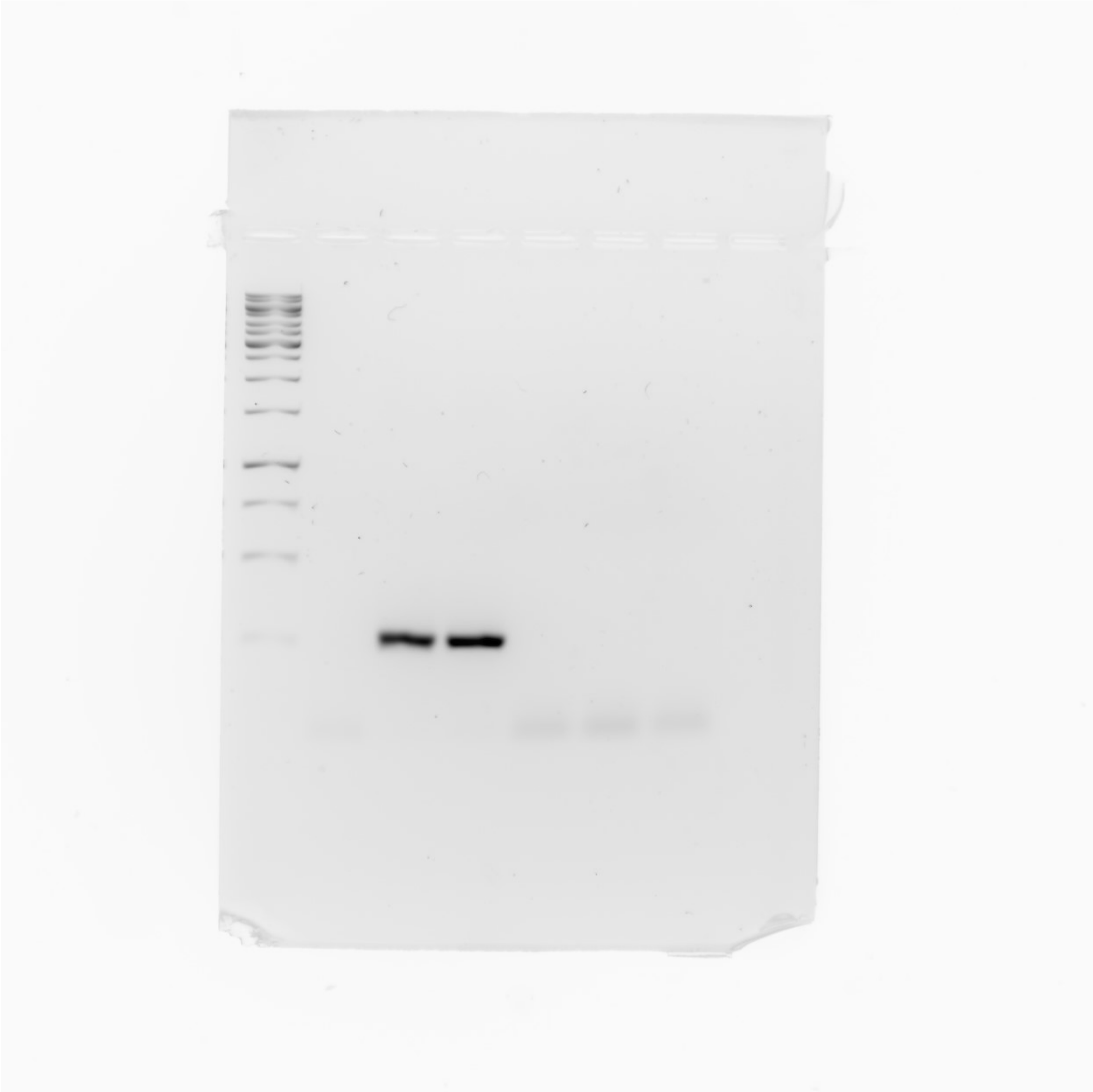

Young L, Sung J, Stacey G, Masters JR. Detection of Mycoplasma in cell cultures. **Nat Protoc**. 2010 May;5(5):929-34. doi: 10.1038/nprot.2010.43. Epub 2010 Apr 22. PMID: 20431538.
