# Supplementary figures and images for: Bone from Healthy Individuals and Patients with CKD Expresses the Sodium-Glucose Co-transporter-2 (SGLT2)
Source: Calcif Tissue Int. 2026 Mar 13;117(1):39. doi: 10.1007/s00223-026-01498-7 (PMC12982296; doi:10.1007/s00223-026-01498-7)

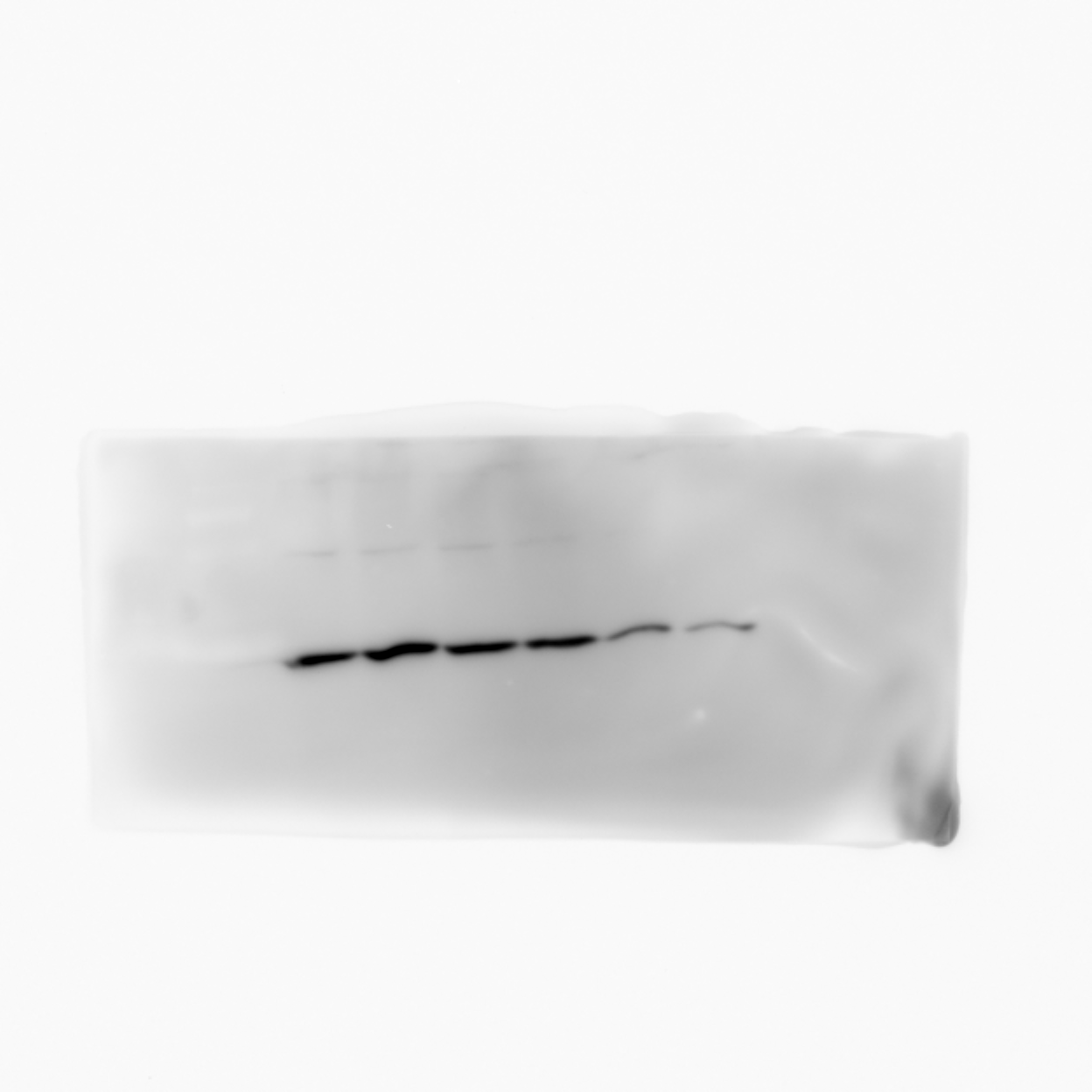

Supplement: Supplementary file 2 — Supplementary file2 (TIF 3042 kb) [file 223_2026_1498_MOESM2_ESM.tif]

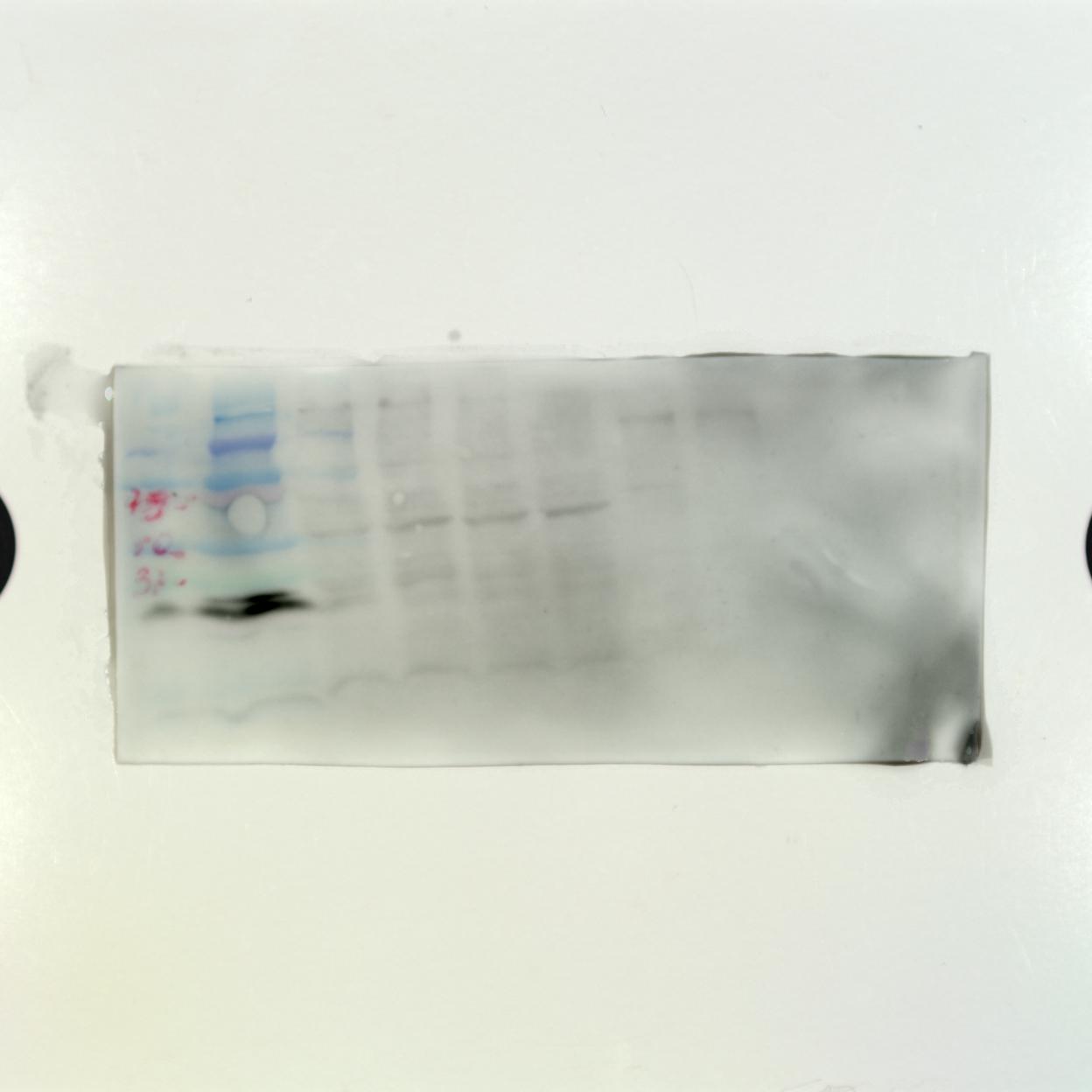

Supplement: Supplementary file 5 — Supplementary file5 (JPG 49 kb) [file 223_2026_1498_MOESM5_ESM.jpg]

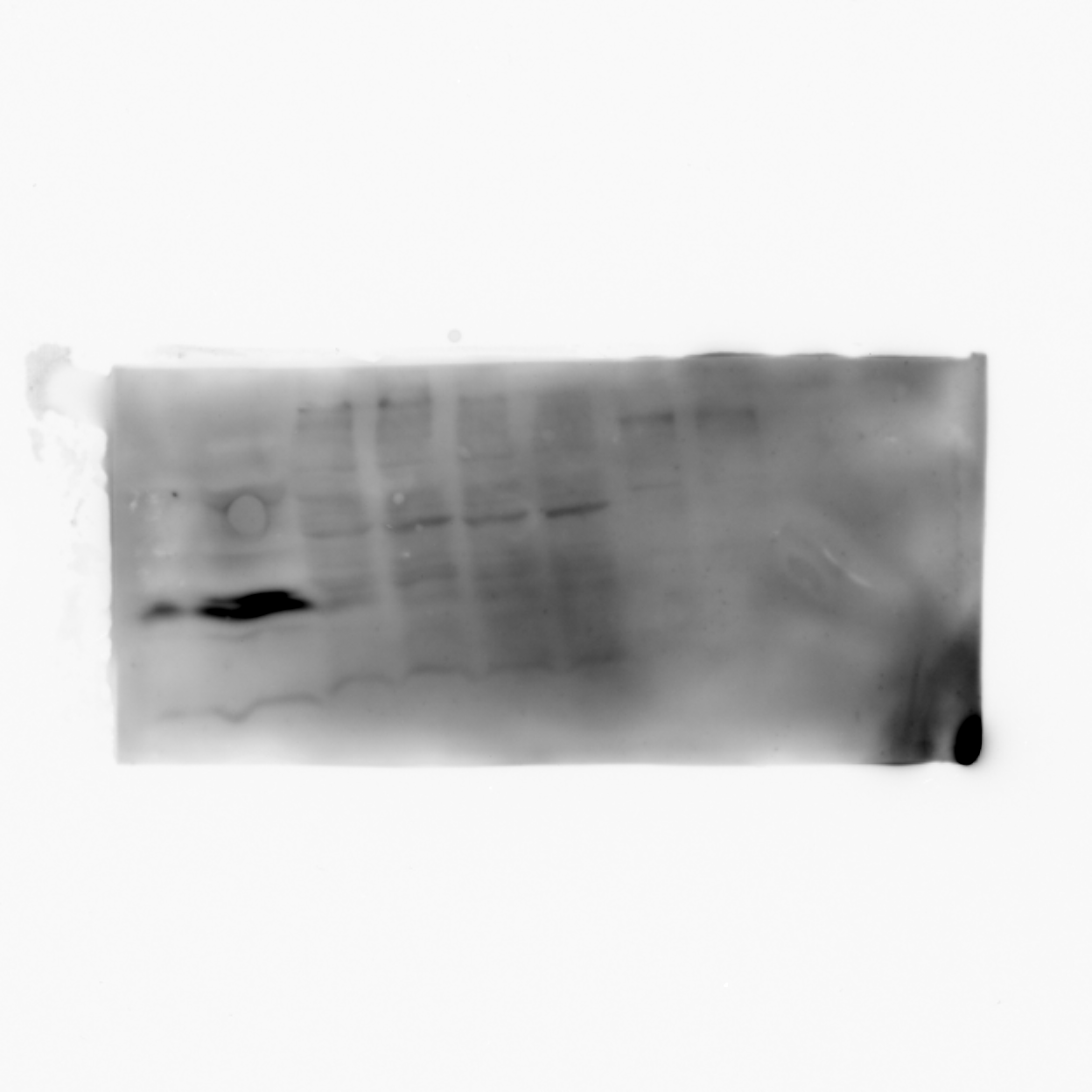

Supplement: Supplementary file 6 — Supplementary file6 (TIF 3042 kb) [file 223_2026_1498_MOESM6_ESM.tif]

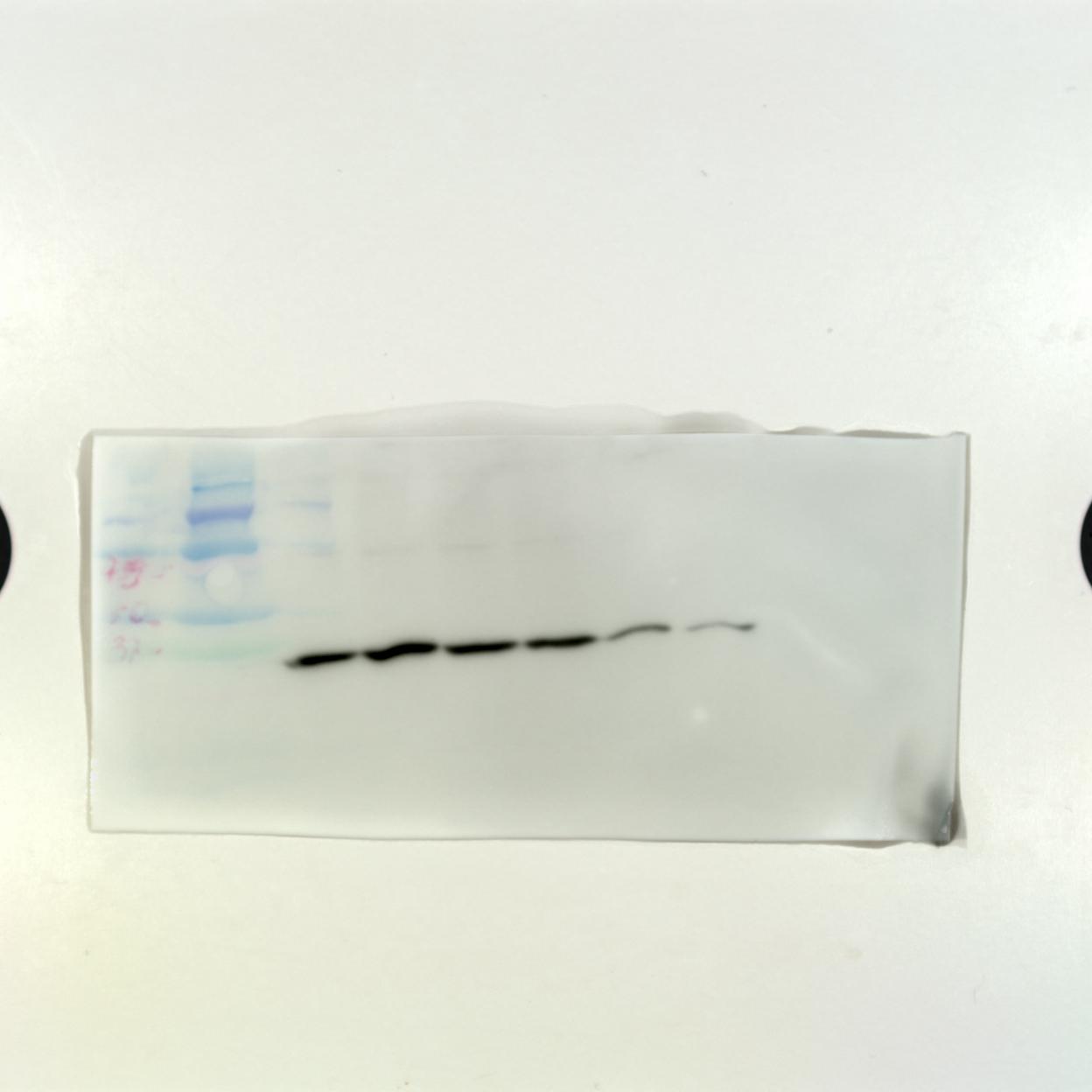

Supplement: Supplementary file 7 — Supplementary file7 (JPG 49 kb) [file 223_2026_1498_MOESM7_ESM.jpg]
