# Supplementary material for: Bone from Healthy Individuals and Patients with CKD Expresses the Sodium-Glucose Co-transporter-2 (SGLT2)
Source: Calcif Tissue Int. 2026 Mar 13;117(1):39. doi: 10.1007/s00223-026-01498-7 (PMC12982296; doi:10.1007/s00223-026-01498-7)
Supplement: Supplementary file 4 — Supplementary file4 (DOCX 14 kb) [file 223_2026_1498_MOESM4_ESM.docx]

**Original files from Western blot**

Below are the uncropped original files used for composing Figure 2 from the main file.

They are here provided in the same order (with and without) the colorimetric marker for reference. SGTL2 antibody code ab37296 and GAPDH antibody code 5174.

The files’ original names are:

1. sglt2 20250703_095352_Ch+Marker.jpg
2. ab37296 20250703_092346-12_Ch.tif
3. 5174 20250703_140308_Ch+Marker.jpg
4. 20250703_134950-12_Ch.tif
